# Proton nuclear magnetic resonance spectroscopy based investigation on propylene glycol toxicosis in a Holstein cow

**DOI:** 10.1186/1751-0147-51-25

**Published:** 2009-06-13

**Authors:** Hanne Christine Bertram, Bent Ole Petersen, Jens Ø Duus, Mogens Larsen, Birgitte-Marie L Raun, Niels Bastian Kristensen

**Affiliations:** 1 Department of Food Science, Faculty of Agricultural Sciences, Aarhus University, P.O. Box 102, DK-5792 Årslev, Denmark; 2 Carlsberg Laboratory, Gamle Carlsberg Vej 10, DK-2500 Valby, Denmark; 3 Department of Animal Health, Welfare and Nutrition, Faculty of Agricultural Sciences, Aarhus University, P.O. Box 50, DK-8830 Tjele, Denmark

## Abstract

**Background:**

It is unknown which metabolites are responsible for propylene glycol (PG)-induced toxicosis, and a better understanding of the underlying mechanisms explaining incidences of abnormal behaviour of dairy cows fed PG is therefore needed.

**Methods:**

The study included three cows of which one developed PG toxicosis. In order to investigate how the metabolism of PG differed in the cow developing toxicosis, proton nuclear magnetic resonance (NMR) spectroscopy was applied on ruminal fluids and blood plasma samples obtained before and after feeding with PG.

**Results:**

PG toxicosis was characterized by dyspnea and ruminal atony upon intake of concentrate containing PG. The oxygen saturation of arterial blood haemoglobin and the oxygen pressure in arterial blood decreased along with the appearance of the clinical symptoms. NMR revealed differences in plasma and ruminal content of several metabolites between the cow responding abnormally to PG and the two control cows.

**Conclusion:**

It is concluded that PG-toxicosis is likely caused by pulmonary vasoconstriction, but no unusual metabolites directly related to induction of this condition could be detected in the plasma or the ruminal fluid.

## Background

Propylene glycol (PG) has been used as a glucogenic feed supplement for ruminants for decades [1]. Metabolism of PG in ruminants involves microbial metabolism in the rumen and hepatic metabolism of products of ruminal fermentation (propanol, propanal, and propionate) as well as PG absorbed to the portal blood [2]. Various application forms of PG are in use: oral drench, oro-ruminal infusion devices, top dressed on feed, mixed into pelleted feeds, and mixed into total mixed rations. Numerous studies report beneficial effects of PG on glucose and fat homeostasis in periparturient dairy cows, for review see [3]. However, reports from practice and sparse reports in the literature describe abnormal behaviour involving observation of shallow breathing, ataxia, salivation, somnolence and depression when adding PG to the feed of dairy cows [3]. In a field trial involving 7 dairy herds, cows were fed either 0, 150, 300 or 450 g PG/d from 20 to 14 d antepartum [4]. In 3 out of the 7 herds approximately 36% of the cows reacted during the first few days of application by signs described as hyperventilation and somnolence.

The present study is based on a test-feeding trial with a pelleted concentrate containing PG, which is under development for use in very early lactation. Feeding this concentrate induced unexpectedly a condition in one out of three cows resembling PG toxicosis and the present study aimed to investigate the metabolites responsible for PG-induced toxicosis using proton nuclear magnetic resonance (NMR) spectroscopy. Since it remains unknown which metabolites are responsible for a PG-induced toxicosis, a non-selective analytical method that detects as many metabolites as possible would be attractive. Proton NMR spectroscopy, which in principle enables detection of all hydrogen-containing molecules, has turned into a commonly applied technique for metabolic profiling of biofluids, among other to identify biochemical changes in response to disease in mammals [5]. In the present study proton NMR spectroscopy was applied on ruminal and blood plasma samples from the cows used in the study.

## Materials and methods

### Animals and feeding

Three Danish Holstein cows (595 ± 42 kg body weight; 22 ± 1 kg milk/d; 276 ± 118 days in milk; 134 ± 14 days after surgery) implanted with a ruminal cannula and permanent indwelling catheters in the hepatic portal vein, mesenteric vein as well as an artery were used in the study. Surgical procedures have been described previously [6,7]. Cows were fed 11 kg/d of a pelleted concentrate (Table 1) and 9 ± 1 kg/d of mixed grass hay (97% dry matter; 59% neutral detergent fiber in dry matter), corresponding to 275 g PG/day. The feed was divided into two equally sized portions fed at 0700 and 1900 h. Cows were milked twice daily and were housed in tie stalls on wood shavings and had free access to water.

**Table 1:** Composition of pelleted concentrate^1^

**Feedstuff**	**Inclusion, % of concentrate (as mixed)**
WeiPass^2^	49
Soya meal	15
Grass meal	10
Sugar beet pulp	10
Molasses, beet	7
Leci-E^3^	3
Propylene glycol	2.5
Sodium bicarbonate	1.5
Mineral mix	1.1
Calcium carbonate	0.5
MetaSmart^4^	0.4
Monocalcium phosphate	0.3

^1 ^The dry matter content was 96.6% and the concentrate contained (% of dry matter): crude protein (N × 6.25), 26; neutral detergent fiber, 19; ash, 8; ether extract 5. The in vitro digestibility of the concentrate was 96.2%.

^2 ^Ruminal protected wheat (Raiffeisen Hauptgenossenschaft Nord AG, Kiel, Germany)

^3 ^Vegetable fat (Leci-E; Evilec, Kolding, Denmark) with 50% rape seed and soybean lecithin and natural α-tocopherol containing (per kg DM): 950 g crude fat, 836 g fatty acids and 2000 mg RRR-α-tocopherol.

^4 ^Isopropyl ester of methionine hydroxyanalog (Adisseo, Antony, France).

The study complied with the Danish Ministry of Justice Law no. 382 (June 10, 1987), Act no. 726 (September 9, 1993) concerning experiments with animals and care of experimental animals.

### Experimental samplings

Each cow was sampled for one day after being fed the experimental diet for 14 d. On sampling days, continuous infusion of *p*-aminohippuric acid (pAH; 29 ± 1 mmol/h) into the mesenteric vein was initiated at 05:30. The pAH infusate was a sterilized 250 mM solution of pAH (4-aminohippuric acid 99%, Acros, Geel, Belgium) adjusted to pH 7.4. Ten sets of ruminal and blood samples were obtained 0.5 h before feeding, and 0.5, 1.5, 2.5, 3.5, 5.0, 6.5, 8.0, 9.5, and 11 h after feeding. Blood was sampled by simultaneously drawing blood from the artery and hepatic portal vein into 20 mL syringes and was immediately transferred to heparin vacuettes (#455051; Greiner Bio-One GmbH, Kremsmuenster, Austria). Plasma was harvested by centrifugation at 3000 *g *for 20 min and stored at -20°C until analysis. Separate blood samples were obtained in 1 mL heparinized syringes for blood gas measurements just before collection of the main blood samples. One extra arterial 1 mL sample was obtained from the cows that reacted to feeding 1 h after feeding. Ruminal fluid was sampled from the ventral ruminal sac using a suction strainer (#RT extended version, Bar Diamond, Parma, ID) and a 50 mL syringe. Ruminal fluid pH was measured immediately after sampling (IQ 150 pH meter; IQ Scientific Instruments Inc., Carlsbad, CA), and a subsample of ruminal fluid was stabilized with 5% meta phosphoric acid and frozen at -20°C.

### Analytical procedures

Blood sampled in 1 mL syringes was immediately taken for blood gas and oximetry analysis (ABL 520, Radiometer A/S, Copenhagen, Denmark).

### NMR spectroscopy

The NMR measurements were performed on a Bruker 800 spectrometer, operating at a ^1^H frequency of 799.40 MHz, and equipped with a 5-mm ^1^H observe TXI cryoprobe (Bruker BioSpin, Rheinstetten, Germany). For both plasma and ruminal samples the NMR measurements were carried out on samples collected 0.5 h before feeding, and 0.5, 1.5, 2.5, 3.5, 5.0 and 8.0 h after feeding. On plasma samples the NMR measurements were carried out at 310 K, while measurements on ruminal fluid samples were carried out at 298 K. Prior to NMR measurements, the samples were thawed, and 500 μl aliquots were mixed with 100 μl D_2_O. Sodium trimethylsilyl- [2,2,3,3-^2^H_4_]-1-propionate (TSP) was added as an internal chemical shift standard (0.10% w/w). The NMR measurements were essentially carried out as described previously described [8]. For ruminal fluid samples ^1^H NMR spectra were obtained using a standard single 90° pulse experiment, while for plasma samples two ^1^H NMR spectra were obtained on each sample; i) a standard one-dimensional spectrum acquired using single 90° pulse experiment, and ii) a one-dimensional spectrum acquired with a Carr-Purcell-Meiboom-Gill (CPMG) delay of 50 ms added in order to attenuate broad signals from high-molecular-weight components. On plasma samples 64 scans were acquired in the CPMG experiment, 32 scans were acquired in the standard spectrum, while 64 scans were acquired on ruminal fluid samples. In all NMR experiments water suppression was achieved by irradiating the water peak during the relaxation delay of 5 s and 16 K data points spanning a spectral width of 13.03 ppm were collected. An exponential line-broadening function of 0.3 Hz was applied to the free induction decay (FID) prior to Fourier transformation (FT). All spectra were referenced to the TSP signal at 0 ppm.

To aid spectral assignment 2D ^1^H-^1^H correlation (DQFCOSY) and 2D ^1^H-^13^C HSQC spectra were recorded on selected ruminal fluid samples using water suppression. The DQFCOSY spectra were acquired with a spectral width of 10000 Hz in both dimensions, 4096 data points, 512 increments with 64 transients per increment and zero filled in the F1 dimension. The HSCQ spectra were acquired with a spectral width of 10000 Hz in the F2 dimension and 30153 Hz in the F1 dimension, a data matrix with a size of 2048 × 512 data points and 32 transients per increment, and the spectra were zero filled in both dimensions.

### Post-processing and multivariate data analysis

Principal component analysis (PCA) was applied to explore any clustering behaviour of the samples using the Unscrambler software version 9.2 (Camo, Oslo, Norway). PCA is an unbiased mathematical algorithm that lowers data dimensionality whilst retaining variation in a large dataset. By identifying directions (principal components) in which variation are at maximum, samples can be explained by a relatively low number of components instead of thousands of variables. Following analysis of the components plots can then be used to identify similarities and differences between samples [9]. The NMR spectra were subdivided into 0.002 ppm integral regions and integrated, and for ruminal fluid spectra the regions 0.5–4.6–10.0 ppm and for plasma spectra the regions 0.5–4.5 and 5.1–10.0 were included in the PCA.

## Results

### Clinical observations

Cows were fed the experimental diet for 14 d prior to sampling and no signs of lack of tolerance to the ration were noticed. On the sampling day, which was designated as the sampling day, two of the cows consumed the entire amount of offered concentrate within 15 min whereas the third cow had consumed approximately half and stopped eating. Three min later the remaining amount of the concentrate was introduced into the rumen via the ruminal cannula. Twelve min after feeding the concentrate directly into the rumen (30 min after feeding) and immediately before starting the second blood sampling the third cow developed severe dyspnea and ruminal atony. The cow remained standing although she was severely affected by the incidence. Two h after feeding the symptoms had completely disappeared and it was observed that the cow started eating hay. By 2.5 h after feeding she was observed drinking water and had apparently completely recovered.

### Oximetry

**Figure 1:**
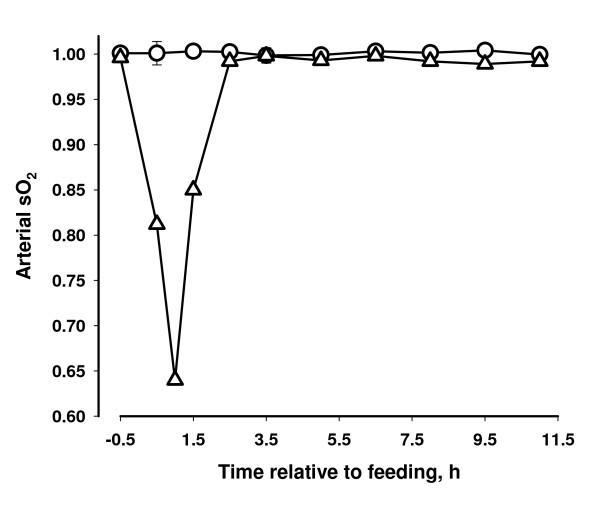
**Oxygen saturation (sO_2_) of arterial blood haemoglobin in two cows that did not show clinical reaction to concentrate containing propylene glycol (circle) and in one cow that developed dyspnea following intake and force feeding with concentrate containing propylene glycol (triangles)**.

**Figure 2:**
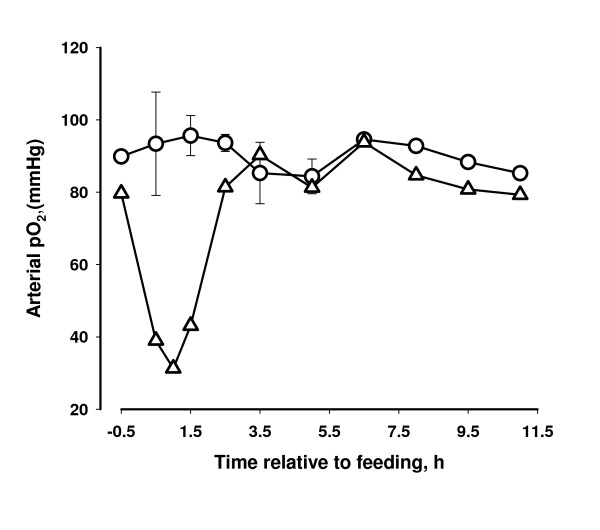
**Oxygen pressure (pO_2_) of arterial blood in two cows that did not show clinical reaction to concentrate containing propylene glycol (circle) and in one cow that developed dyspnea following intake and force feeding with concentrate containing propylene glycol (triangles)**.

**Figure 3:**
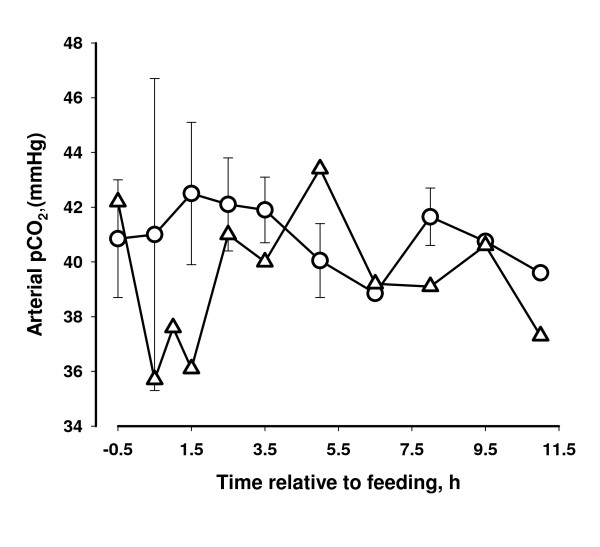
**Carbon dioxide pressure (pCO_2_) of arterial blood in two cows that did not show clinical reaction to concentrate containing propylene glycol (circle) and in one cow that developed dyspnea following intake and force feeding with concentrate containing propylene glycol (triangles)**.

One h after feeding the oxygen saturation of arterial blood haemoglobin of the affected cow decreased to 0.64, and the curve reflects the observed clinical condition of the cow. The oxygen saturation of the two other cows did not change following feeding (Figure 1). The affected cow was hypoxemic with a decrease in pO_2 _(oxygen pressure) of arterial blood following the same pattern as the oxygen saturation (Figure 2). Only a slight decrease in pCO_2 _(carbon dioxide pressure) was observed for the affected cow (Figure 3).

### Plasma analyses

**Figure 4:**
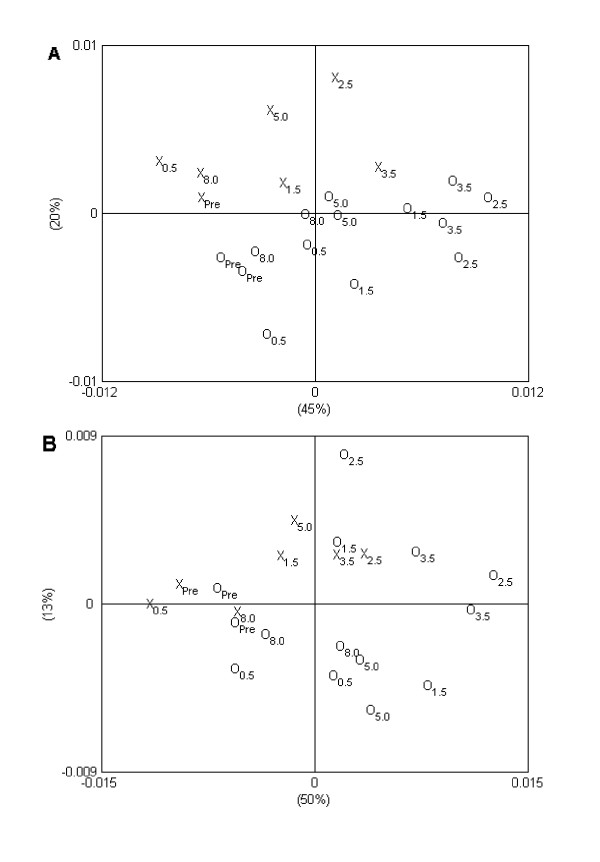
**Principal component analysis score plot showing the two first principal components (PCs) for (A) arterial, and (B) portal plasma samples**. Labels on axes show how much of the variation in the data that is explained by the PCs. Sample id: The two normal cows are represented by 'O', while the cow responding abnormally to PG is represented by 'X'. Subscript in sample id shows sampling time in hours after feeding, "pre" representing samples obtained before feeding.

In order to investigate the main variations in the serial plasma metabolite profiles, PCAs were performed on the obtained NMR spectra, and score plots are shown in Figure 4. For both arterial plasma (Figure 4a) and portal plasma samples (Figure 4b) the first principal component (PC1) appeared to describe a manifest effect of sampling time, as a clear movement of samples along PC1 as function of sampling time was observed. The largest difference was observed between samples obtained before feeding and samples obtained 2.5–3.5 h after feeding, while samples obtained 5.0 and 8.0 h after feeding shifted back towards the samples obtained before feeding. Especially for arterial plasma it was clear that the second principal component (PC2) explained the variation between control samples and samples from the cow responding abnormally to PG, as the samples from the cow responding abnormally to PG in general were characterized by higher PC2 score values (Figure 4a). This revealed that irrespective of sampling time after feeding, the plasma metabolite profile of the cow responding abnormally to PG administration differed from the plasma metabolite profile of the two control cows.

**Figure 5:**
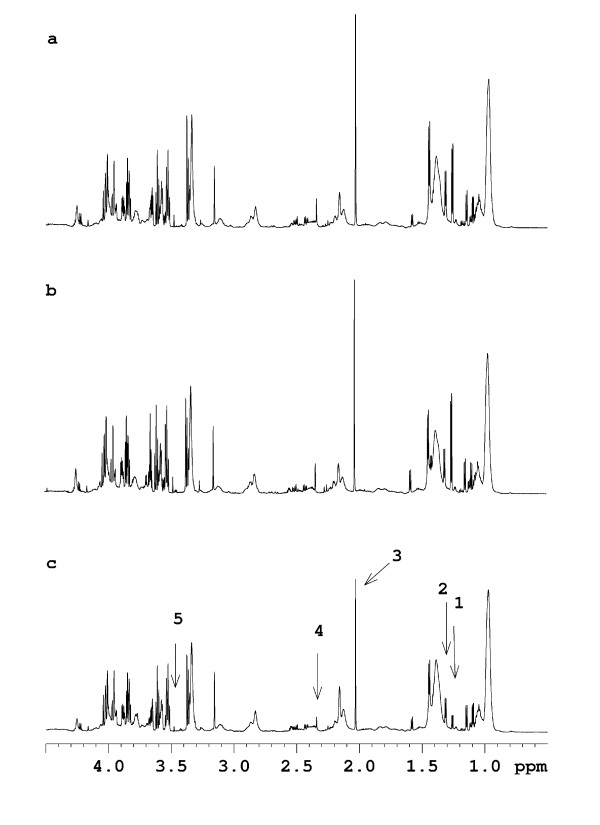
**^1^H CPMG NMR spectra obtained on arterial plasma samples obtained 0.5 h after feeding from the two control cows (a+b) and the cow responding abnormally to propylene glycol (PG)-induced toxicosis (c)**. The arrows indicate the signals that are lower in intensity in the cow responding abnormally to PG supplementation compared with the two control cows: 1: isopropanol/isobutyrate, 2: β-hydroxybutyrate, 3:acetate, 4: acetoacetate, and 5: acetone.

For a more comprehensive analysis of metabolic differences at distinct sampling times, the ^1^H CPMG NMR metabolite profiles obtained on plasma samples obtained from the different cows but at the same sampling time were analysed. Figure 5 shows the ^1^H CPMG NMR metabolite profile obtained on arterial plasma samples obtained 0.5 h after feeding with PG. The same metabolites were present in all arterial plasma samples. However, the plasma spectrum of the cow that responded abnormally to PG was characterized by lower intensities of signals assigned to isopropanol and isobutyrate (1.17 ppm), β-hydroxybuturate (1.22 ppm), acetate (1.93 ppm), acetone (2.22 ppm) and acetoacetate (3.38 ppm) compared with the plasma spectra from the two control cows (Figure 5). In NMR spectra of arterial plasma samples obtained 1.5 h or later after feeding the difference in the intensity of the signal assigned to acetate (1.93 ppm) between cows had disappeared, and the differences in the intensities of the other metabolites found to differ 0.5 h after feeding likewise diminished and disappeared with increasing time after feeding. An identical pattern was observed in the NMR spectra of portal plasma samples (data not shown). Independent of sampling time and plasma type, a higher intensity of the characteristic broad signals arising from lipids (~0.9, 1.25 and 2.02 ppm) was observed in ^1^H NMR spectra of plasma from the cow responding abnormally to PG compared with the two control cows.

### Ruminal fluid

**Figure 6:**
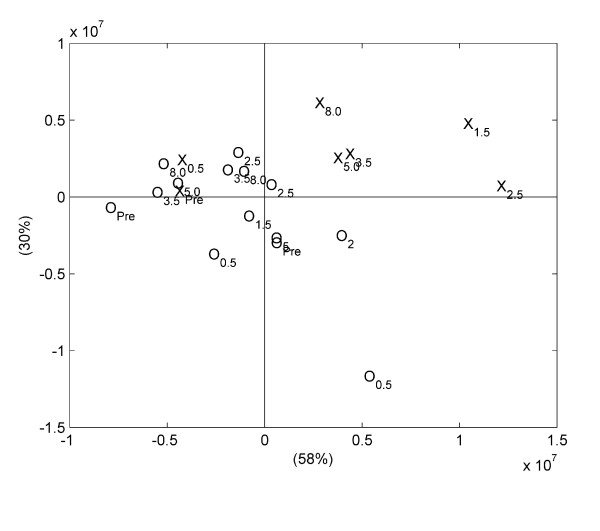
**Principal component analysis score plot showing the two first principal components for ruminal fluid samples**. Labels on axes show how much of the variation in the data that is explained by the PCs. Sample id: The two normal cows are represented by 'O', while the cow responding abnormally to propylene glycol is represented by 'X'. Subscript in sample id shows sampling time in hours after feeding, "pre" representing samples obtained before feeding.

**Figure 7:**
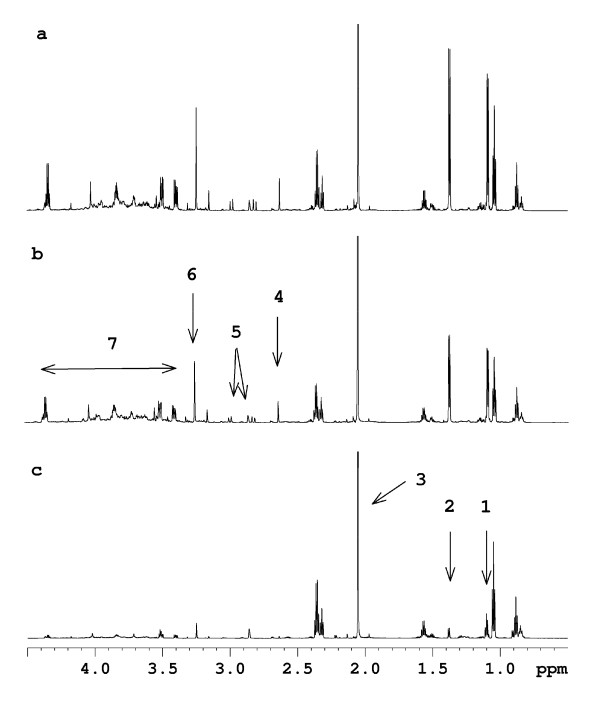
**^1^H NMR spectra obtained on ruminal fluid samples obtained 0.5 h after feeding from the two control cows (a+b) and the cow responding abnormally to propylene glycol (PG) (c)**. The arrows indicate the signals that are lower in intensity in the cow responding abnormally to PG supplementation compared with the two control cows: 1: isopropanol/isobutyrate, 2: lactate, 3: acetate, 4: acetone, 5: citrate, 6: methyl acetate, 7: various smaller esters and alcohols.

**Figure 8:**
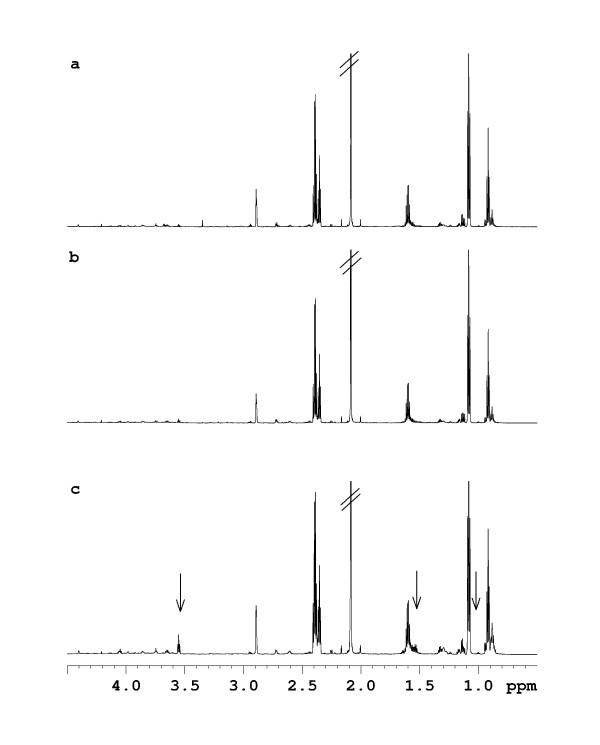
**^1^H NMR spectra obtained on ruminal fluid samples obtained 2.5 h after feeding from the two control cows (a+b) and the cow responding abnormally to propylene glycol (PG) (c)**. The arrows show signals that have been assigned to propanol. The signals from propanol are considerably higher in intensity in the cow responding abnormally to PG compared with the two control cows.

For an elucidation of the main variations in the serial ruminal fluid metabolite profiles, PCA was performed on the NMR spectra. Noticeably, a clear separation of all ruminal fluid samples obtained 1.5 h after feeding or later from the cow responding abnormally to PG was seen along the first component (Figure 6). For a more comprehensive analysis of metabolic differences at distinct sampling times, the ^1^H NMR metabolite profiles obtained on ruminal fluid samples obtained from the different cows but at the same sampling time were analysed. Comparison of the NMR spectra of ruminal fluid samples obtained 0.5 h after feeding revealed significantly lower intensities of signals assigned to isopropanol and isobutyrate (1.18 ppm), lactate (1.38 ppm and 4.35 ppm), acetate (2.08 ppm), acetone (2.63 ppm) and citrate (2.88, 2.90, 3.04 and 3.06 ppm) in the metabolite profile of the cow responding abnormally to PG compared with the two control cows (Figure 7). In addition, the NMR spectrum of the ruminal fluid sample obtained 0.5 h after feeding from the cow responding abnormally to PG was also characterized by lower intensities of several small peaks in the region ~3.4–4.4 ppm, which is tentatively assigned to various small esters and alcohols. In addition, the NMR spectrum of the ruminal fluid sample obtained 0.5 h after feeding from the cow responding abnormally to PG was characterised by a lower intensity of a signal at 3.3 ppm, which is tentatively assigned to the methyl group in methyl acetate. The NMR spectra of ruminal fluid samples obtained 1.5 h and 2.5 h after feeding showed a pronounced increase in intensities of signals assigned to propanol (0.90, 1.55 and 3.55 ppm) for the cow responding abnormally to PG compared with the two control cows (Figure 8).

## Discussion

Examples of PG-induced toxicosis have been reported in ruminants [4] and other animals [10-13]. However, these animals have rarely been further examined, and the molecular mechanisms causing the abnormal response are unknown. In the present study PG was applied in a pelleted concentrate and the effects of PG cannot be separated from the effects of other ingredients as such. However, the observed clinical signs of the cow that responded badly to the ration are in good agreement with the reports from farmers, extension personnel, and veterinarians on reaction to introduction of PG containing concentrates in dairy herds. Accordingly, evidence exists that the incidence under investigation is PG toxicosis; however, it cannot be ruled out at present if other dietary components contributed to the incidence.

The oximetry data together with the clinical picture suggests that the hypoxia of the affected cow was caused by decreased gas exchange between the pulmonary alveoli and the blood and not caused by changes in oxygen affinity of haemoglobin (both oxygen tension and saturation decreased in parallel) and the breathing of the cow appeared to be both of high frequency and with full depth. The condition of the cow could be caused by pulmonary vasoconstriction (brisket disease) similar to the response of cattle seen at high altitude [14]. However it is unlikely that PG itself induced the pulmonary vasoconstriction because high plasma concentrations of PG have been attained in previous studies without any apparent effects on the cows [2,15].

The present investigation is the first to report the use of ^1^H NMR-based metabolic profiling in the study of PG metabolism and toxicity. High-resolution ^1^H NMR spectra could be obtained in both ruminal fluid and plasma samples, enabling the detection of several metabolites. PCA on the serial metabolite profiles revealed differences between the abnormal cow and the two control cows both in ruminal fluid, arterial and portal plasma samples. Accordingly, data indicated that the PG-induced toxicosis was associated with a different metabolic response to the feeding. Further analysis of the metabolite profiles of the ruminal samples revealed that this abnormal response was reflected in lower contents of isopropanol, isobutyrate, lactate, acetate, acetone, citrate and some unidentified, smaller esters and alcohols in the ruminal fluid shortly (0.5 h) after feeding. However, the lower concentrations as compared with the control cows could indicate a general decrease in microbial fermentation in the rumen. Later in the course corresponding to 1.5–2.5 h after feeding, the ^1^H NMR spectra of ruminal fluid from the cow responding abnormally to the ration were characterized by considerably higher intensities of signals ascribed to propanol. However, also propanol has previously been observed in high ruminal and plasma concentrations without affecting the cows [2].

^1^H NMR spectroscopy of the plasma samples revealed that the cow that responded abnormally to PG was characterized by a lower content of isopropanol, isobutyrate, β-hydroxybuturate, acetate, acetone and acetoacetate shortly after feeding (0.5 h) compared with the respective arterial and portal plasma samples from the two control cows. However, the lower concentrations as compared with the control cows is likely caused by reduced fermentation activity in the rumen in combination with reduced absorption because of ruminal atony.

In addition to these differences in the concentration of low-molecular-weight metabolites, the ^1^H NMR spectra also revealed a higher lipid content in plasma of the cow responding abnormally to PG compared with the control cows. This is very unlikely an effect of PG, as it was also present in the samples obtained before feeding. In contrast, the higher lipid content in plasma of the cow responding abnormally to PG probably reflects a natural variation. It remains unknown if the higher plasma lipid content is associated with a higher susceptibility for development of PG toxicosis.

Beside lower concentrations of common metabolites/fermentation products in ruminal fluid and plasma samples immediately after feeding, the NMR spectra could not reveal the presence of any "extraordinary" or unusual metabolites in the biofluids of the cow developing toxicosis. It has recently been suggested that sulphur-containing compounds produced during fermentation of PG could be the cause of side effects [16]. We observed no indications of the presence of sulphur-containing compounds in the NMR spectra. Plausible explanations for the lack of detection in the NMR spectra exist, as the compounds are volatile or present in concentrations below the detection limit of NMR. It has recently been established that H_2_S is an important signalling substance in hypoxic vasoconstriction in vertebrates including cattle [17]. Therefore sulphur compounds or specifically H_2_S appear as promising candidates for explaining the link between PG application to the rumen and the dyspnea of the cow.

## Conclusion

The present study showed that the symptoms of PG-toxicosis are likely to be caused by pulmonary vasoconstriction, however, it was not possible to identify the metabolites inducing the response by use of ^1^H NMR spectroscopy.

## Competing interests

The authors declare that they have no competing interests.

## Authors' contributions

HCB carried out the NMR measurements, analysis and interpretation of NMR data, and drafted the manuscript. BP and JD participated in the NMR measurements, analysis and interpretation of NMR data. NBK and ML were responsible for the experimental part carried out on the cows and helped substantially to draft the manuscript. NBK, ML and BMR all participated in the observational study, the oximetric measurements and sampling. All authors read and approved the final manuscript.
